# Bis(4-chloro­pyridine){2,2′-[ethane-1,2-diylbis(nitrilo­methyl­idyne)]diphenolato}cobalt(III) perchlorate methanol monosolvate

**DOI:** 10.1107/S1600536810047793

**Published:** 2010-11-24

**Authors:** Daopeng Zhang

**Affiliations:** a College of Chemical Engineering, Shandong University of Technology, Zibo 255049, People’s Republic of China

## Abstract

In the title complex, [Co(C_16_H_14_N_2_O_2_)(C_5_H_4_ClN)_2_]ClO_4_·CH_3_OH, the Co^III^ ion is in a slightly distorted octa­hedral CoN_4_O_2_ coordination environment with the two 4-chloro­pyridine ligands in a *trans* arrangement.

## Related literature

For related structures, see: Chen (2008 ▶); Kitaura *et al.* (1987 ▶); Shi *et al.* (1995 ▶); Zhou (2009 ▶).
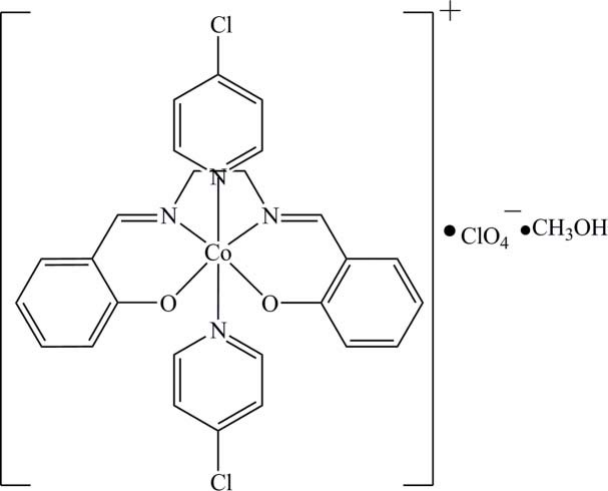


## Experimental

### 

#### Crystal data

[Co(C_16_H_14_N_2_O_2_)(C_5_H_4_ClN)_2_]ClO_4_·CH_4_O
*M* *_r_* = 683.80
Triclinic, 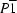

*a* = 9.0244 (12) Å
*b* = 11.2625 (16) Å
*c* = 15.052 (2) Å
α = 92.757 (2)°
β = 103.843 (2)°
γ = 95.396 (2)°
*V* = 1474.9 (3) Å^3^
*Z* = 2
Mo *K*α radiation
μ = 0.91 mm^−1^
*T* = 293 K
0.31 × 0.29 × 0.25 mm

#### Data collection

Bruker APEXII CCD area-detector diffractometer
Absorption correction: multi-scan (*SADABS*; Sheldrick, 2008*a* ▶) *T*_min_ = 0.767, *T*_max_ = 0.805
7378 measured reflections
5144 independent reflections
4213 reflections with *I* > 2σ(*I*)
*R*_int_ = 0.016

#### Refinement

*R*[*F* ^2^ > 2σ(*F* ^2^)] = 0.052
*wR*(*F* ^2^) = 0.160
*S* = 1.05
5144 reflections
380 parameters
H-atom parameters constrained
Δρ_max_ = 1.21 e Å^−3^
Δρ_min_ = −0.49 e Å^−3^

### 

Data collection: *APEX2* (Bruker, 2004 ▶); cell refinement: *SAINT-Plus* (Bruker, 2001 ▶); data reduction: *SAINT-Plus*; program(s) used to solve structure: *SHELXS97* (Sheldrick, 2008*b* ▶); program(s) used to refine structure: *SHELXL97* (Sheldrick, 2008*b* ▶); molecular graphics: *SHELXTL* (Sheldrick, 2008*b* ▶); software used to prepare material for publication: *SHELXTL*.

## Supplementary Material

Crystal structure: contains datablocks I, global. DOI: 10.1107/S1600536810047793/hg2742sup1.cif

Structure factors: contains datablocks I. DOI: 10.1107/S1600536810047793/hg2742Isup2.hkl

Additional supplementary materials:  crystallographic information; 3D view; checkCIF report

## Figures and Tables

**Fig. 1.**
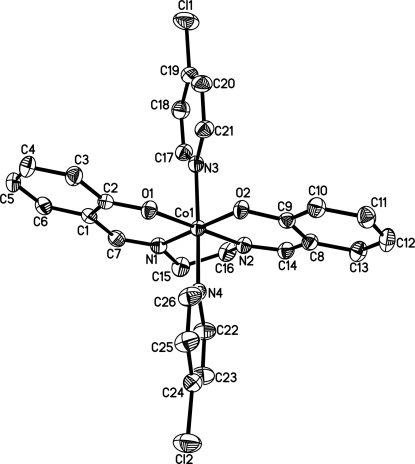
The cation of the title complex with the atom-labelling scheme. Displacement ellipsoids are drawn at the 30% probability level. All the H atoms, the balanced ClO4- anion and the solvent methanol are not shown.

## supplementary crystallographic information

### Comment

Tetradentate Schiff-base ligands, due to their excellent chelating ability for metal atoms, have been widely used to synthsize transition metal complexes. Here, we report the crystal structure of a Co^III^ complex based on tetradentate Schiff base ligand *N*,*N*'-bis(salicylidene)-1,2-diphenyl-1,2-ethanediamine.

The cation structure of the title complex is shown in Fig. 1. The Co^III^ ion is six coordinated by a N_4_O_2_ unit, in which the four equational sites are occupied by two N atoms and two O atoms from the tetradentate Schiff base ligand and the two axial sites are occupied by the N atoms of two 4-chloro-pyridine ligands, therefore forming a slightly distorted octahedral coordination environment. The Co—O, Co—N_Schiff-base_ and Co—N_pyridine_bond lengths are 1.891 (2), 1.898 (2),1.892 (3), 1.897 (3), 1.977 (3) and 1.995 (3)A%, respectively, which are all comparable to the corresponding bond lengths found in the previously reported Co^III^ Schiff-base complexes (Chen, 2008; Kitaura, *et al.*, 1987; Shi, *et al.*, 1995; Zhou, 2009).

### Experimental

The synthesis of the title complex was carried out by mixing Co(ClO_4_)_2_.6H_2_O (0.1 mmol, 36.6 mg), 4-chloro-pyridine (0.2 mm mol, 22.8 mg) and the Schiff-base ligand (0.1 mmol, 26.8 mg) in methanol. After the mixture was stirred for about half an hour at room temperature in air, it was filtered, and the filtrate was allowed to partially evaporate for one week to produce crystals suitable for X-ray diffraction with an yield about 51%. Anal. Calcd for C_27_H_26_Cl_3_CoN_4_O_7_: C, 47.42; H, 3.83; N, 8.19. Found: C, 47.54; H, 3.75; N, 8.095. Main IR bands (cm^-1^): 3020 (s, *C*—H),1618 (m, C=N), 1093 (s, Cl=O).

### Refinement

All the H atoms bonded to the C atoms were placed using the HFIX commands in *SHELXL-97* with C—H distances of 0.93 and 0.96 Å, and were allowed for as riding atoms with *U*_iso_(H) = 1.2*U*_eq_(C) or *U*_iso_(H) = 1.5*U*_eq_(C), respectively. For the H atoms bonded to O atom, it was found from difference Fourier maps with the bond lengths restrained to 0.82 Å, and was allowed for as riding atoms with *U*_iso_(H) = 1.5*U*_eq_(O).

### Crystal data

**Table:**

[Co(C_16_H_14_N_2_O_2_)(C_5_H_4_ClN)_2_]ClO_4_·CH_4_O	*Z* = 2
*M**_r_* = 683.80	*F*(000) = 700
Triclinic, *P*1	*D*_x_ = 1.540 Mg m^−^^3^
Hall symbol: -P 1	Mo *K*α radiation, λ = 0.71073 Å
*a* = 9.0244 (12) Å	Cell parameters from 1564 reflections
*b* = 11.2625 (16) Å	θ = 2.7–27.9°
*c* = 15.052 (2) Å	µ = 0.91 mm^−^^1^
α = 92.757 (2)°	*T* = 293 K
β = 103.843 (2)°	Block, red-brown
γ = 95.396 (2)°	0.31 × 0.29 × 0.25 mm
*V* = 1474.9 (3) Å^3^	

### Data collection

**Table:**

Bruker APEXII CCD area-detector diffractometer	5144 independent reflections
Radiation source: fine-focus sealed tube	4213 reflections with *I* > 2σ(*I*)
graphite	*R*_int_ = 0.016
φ and ω scans	θ_max_ = 25.0°, θ_min_ = 1.4°
Absorption correction: multi-scan (*SADABS*; Sheldrick, 2008*a*)	*h* = −10→10
*T*_min_ = 0.767, *T*_max_ = 0.805	*k* = −12→13
7378 measured reflections	*l* = −12→17

### Refinement

**Table:**

Refinement on *F*^2^	Primary atom site location: structure-invariant direct methods
Least-squares matrix: full	Secondary atom site location: difference Fourier map
*R*[*F*^2^ > 2σ(*F*^2^)] = 0.052	Hydrogen site location: inferred from neighbouring sites
*wR*(*F*^2^) = 0.160	H-atom parameters constrained
*S* = 1.05	*w* = 1/[σ^2^(*F*_o_^2^) + (0.0871*P*)^2^ + 1.6441*P*] where *P* = (*F*_o_^2^ + 2*F*_c_^2^)/3
5144 reflections	(Δ/σ)_max_ < 0.001
380 parameters	Δρ_max_ = 1.21 e Å^−^^3^
0 restraints	Δρ_min_ = −0.49 e Å^−^^3^

### Special details

**Table:**

Geometry. All e.s.d.'s (except the e.s.d. in the dihedral angle between two l.s. planes) are estimated using the full covariance matrix. The cell e.s.d.'s are taken into account individually in the estimation of e.s.d.'s in distances, angles and torsion angles; correlations between e.s.d.'s in cell parameters are only used when they are defined by crystal symmetry. An approximate (isotropic) treatment of cell e.s.d.'s is used for estimating e.s.d.'s involving l.s. planes.
Refinement. Refinement of *F*^2^ against ALL reflections. The weighted *R*-factor *wR* and goodness of fit *S* are based on *F*^2^, conventional *R*-factors *R* are based on *F*, with *F* set to zero for negative *F*^2^. The threshold expression of *F*^2^ > σ(*F*^2^) is used only for calculating *R*-factors(gt) *etc*. and is not relevant to the choice of reflections for refinement. *R*-factors based on *F*^2^ are statistically about twice as large as those based on *F*, and *R*- factors based on ALL data will be even larger.

### Fractional atomic coordinates and isotropic or equivalent isotropic displacement parameters (Å^2^)

**Table:**

	*x*	*y*	*z*	*U*_iso_*/*U*_eq_	
Co1	0.86908 (5)	0.11688 (4)	0.27546 (3)	0.03389 (18)	
Cl1	0.7528 (2)	−0.42638 (12)	0.38879 (11)	0.0922 (5)	
Cl2	1.12614 (17)	0.66668 (11)	0.25101 (11)	0.0786 (4)	
Cl3	0.18397 (16)	0.27636 (12)	0.02468 (9)	0.0665 (3)	
O1	1.0710 (3)	0.1026 (2)	0.34504 (16)	0.0395 (6)	
O2	0.8300 (3)	0.1686 (2)	0.38825 (16)	0.0390 (6)	
O3	0.0318 (7)	0.2275 (8)	−0.0063 (5)	0.186 (3)	
O4	0.2366 (8)	0.3413 (8)	−0.0360 (5)	0.178 (3)	
O5	0.2650 (11)	0.1777 (7)	0.0309 (7)	0.222 (4)	
O6	0.2264 (10)	0.3178 (9)	0.1133 (4)	0.205 (4)	
O7	0.5606 (10)	0.2638 (9)	0.9505 (7)	0.221 (5)	
H7	0.5417	0.2768	1.0005	0.332*	
N1	0.9058 (4)	0.0653 (3)	0.1619 (2)	0.0401 (7)	
N2	0.6671 (3)	0.1307 (3)	0.2060 (2)	0.0374 (7)	
N3	0.8215 (3)	−0.0521 (3)	0.3026 (2)	0.0384 (7)	
N4	0.9340 (3)	0.2864 (3)	0.2621 (2)	0.0390 (7)	
C1	1.1544 (4)	−0.0002 (3)	0.2244 (3)	0.0397 (8)	
C2	1.1695 (4)	0.0401 (3)	0.3167 (2)	0.0357 (8)	
C3	1.2979 (4)	0.0125 (4)	0.3825 (3)	0.0471 (9)	
H3	1.3099	0.0375	0.4438	0.056*	
C4	1.4067 (5)	−0.0514 (4)	0.3574 (3)	0.0563 (11)	
H4	1.4909	−0.0687	0.4022	0.068*	
C5	1.3924 (5)	−0.0902 (4)	0.2664 (3)	0.0554 (11)	
H5	1.4667	−0.1327	0.2502	0.066*	
C6	1.2690 (5)	−0.0655 (4)	0.2015 (3)	0.0511 (10)	
H6	1.2590	−0.0918	0.1407	0.061*	
C7	1.0261 (5)	0.0194 (4)	0.1520 (3)	0.0448 (9)	
H7A	1.0303	−0.0031	0.0924	0.054*	
C8	0.5817 (4)	0.2362 (3)	0.3251 (3)	0.0392 (8)	
C9	0.7113 (4)	0.2225 (3)	0.3977 (2)	0.0358 (8)	
C10	0.7089 (5)	0.2673 (4)	0.4864 (3)	0.0456 (9)	
H10	0.7915	0.2589	0.5354	0.055*	
C11	0.5884 (5)	0.3226 (4)	0.5022 (3)	0.0516 (10)	
H11	0.5900	0.3504	0.5616	0.062*	
C12	0.4637 (5)	0.3381 (4)	0.4310 (3)	0.0577 (11)	
H12	0.3831	0.3773	0.4423	0.069*	
C13	0.4607 (5)	0.2947 (4)	0.3435 (3)	0.0509 (10)	
H13	0.3768	0.3043	0.2957	0.061*	
C14	0.5654 (4)	0.1835 (3)	0.2337 (3)	0.0413 (9)	
H14	0.4729	0.1884	0.1912	0.050*	
C15	0.7786 (5)	0.0764 (4)	0.0805 (3)	0.0520 (11)	
H15A	0.7719	0.0110	0.0348	0.062*	
H15B	0.7967	0.1511	0.0533	0.062*	
C16	0.6310 (5)	0.0731 (4)	0.1120 (3)	0.0495 (10)	
H16A	0.5571	0.1153	0.0711	0.059*	
H16B	0.5870	−0.0090	0.1114	0.059*	
C17	0.7805 (5)	−0.1467 (4)	0.2413 (3)	0.0454 (9)	
H17	0.7667	−0.1333	0.1795	0.055*	
C18	0.7576 (5)	−0.2624 (4)	0.2642 (3)	0.0541 (11)	
H18	0.7278	−0.3253	0.2192	0.065*	
C19	0.7798 (5)	−0.2828 (4)	0.3555 (3)	0.0534 (11)	
C20	0.8241 (6)	−0.1875 (4)	0.4200 (3)	0.0570 (11)	
H20	0.8399	−0.1992	0.4822	0.068*	
C21	0.8445 (5)	−0.0751 (4)	0.3914 (3)	0.0470 (9)	
H21	0.8760	−0.0113	0.4356	0.056*	
C22	0.8877 (5)	0.3475 (4)	0.1886 (3)	0.0533 (11)	
H22	0.8144	0.3090	0.1388	0.064*	
C23	0.9419 (6)	0.4633 (4)	0.1823 (3)	0.0620 (12)	
H23	0.9067	0.5022	0.1294	0.074*	
C24	1.0492 (5)	0.5210 (4)	0.2553 (3)	0.0522 (10)	
C25	1.0946 (5)	0.4621 (4)	0.3331 (3)	0.0609 (12)	
H25	1.1642	0.5003	0.3846	0.073*	
C26	1.0355 (5)	0.3463 (4)	0.3333 (3)	0.0548 (11)	
H26	1.0676	0.3066	0.3862	0.066*	
C27	0.6289 (12)	0.3621 (9)	0.9273 (7)	0.150 (4)	
H27A	0.6190	0.4289	0.9666	0.225*	
H27B	0.5819	0.3752	0.8647	0.225*	
H27C	0.7357	0.3537	0.9335	0.225*	

### Atomic displacement parameters (Å^2^)

**Table:**

	*U*^11^	*U*^22^	*U*^33^	*U*^12^	*U*^13^	*U*^23^
Co1	0.0333 (3)	0.0412 (3)	0.0266 (3)	0.0087 (2)	0.00415 (19)	0.0042 (2)
Cl1	0.1540 (16)	0.0488 (7)	0.0981 (11)	0.0199 (8)	0.0718 (11)	0.0204 (7)
Cl2	0.0851 (9)	0.0531 (7)	0.0944 (10)	−0.0081 (6)	0.0192 (8)	0.0193 (7)
Cl3	0.0745 (8)	0.0669 (8)	0.0580 (7)	0.0084 (6)	0.0148 (6)	0.0113 (6)
O1	0.0356 (13)	0.0489 (15)	0.0327 (13)	0.0113 (11)	0.0038 (10)	0.0019 (11)
O2	0.0367 (13)	0.0500 (15)	0.0303 (13)	0.0143 (11)	0.0048 (10)	0.0017 (11)
O3	0.092 (4)	0.272 (9)	0.165 (6)	−0.034 (5)	−0.003 (4)	0.019 (6)
O4	0.154 (6)	0.236 (8)	0.141 (5)	−0.011 (5)	0.026 (4)	0.103 (5)
O5	0.214 (8)	0.129 (6)	0.277 (10)	0.069 (6)	−0.044 (7)	−0.029 (6)
O6	0.208 (8)	0.300 (11)	0.093 (4)	0.056 (7)	0.013 (5)	−0.048 (5)
O7	0.202 (8)	0.219 (9)	0.301 (12)	0.090 (7)	0.123 (8)	0.146 (9)
N1	0.0430 (17)	0.0515 (19)	0.0262 (15)	0.0089 (15)	0.0073 (13)	0.0070 (13)
N2	0.0361 (16)	0.0439 (17)	0.0302 (15)	0.0077 (13)	0.0028 (12)	0.0022 (13)
N3	0.0360 (16)	0.0485 (18)	0.0307 (16)	0.0098 (14)	0.0061 (13)	0.0040 (14)
N4	0.0352 (16)	0.0464 (18)	0.0351 (16)	0.0082 (13)	0.0062 (13)	0.0068 (14)
C1	0.041 (2)	0.042 (2)	0.041 (2)	0.0057 (16)	0.0176 (17)	0.0097 (16)
C2	0.0358 (19)	0.0339 (18)	0.0392 (19)	0.0043 (15)	0.0116 (15)	0.0068 (15)
C3	0.039 (2)	0.056 (2)	0.047 (2)	0.0102 (18)	0.0079 (17)	0.0071 (19)
C4	0.036 (2)	0.066 (3)	0.068 (3)	0.017 (2)	0.009 (2)	0.013 (2)
C5	0.041 (2)	0.055 (3)	0.079 (3)	0.0165 (19)	0.027 (2)	0.014 (2)
C6	0.054 (2)	0.054 (2)	0.054 (2)	0.011 (2)	0.028 (2)	0.009 (2)
C7	0.053 (2)	0.053 (2)	0.0327 (19)	0.0087 (19)	0.0177 (17)	0.0069 (17)
C8	0.0362 (19)	0.038 (2)	0.044 (2)	0.0060 (15)	0.0088 (16)	0.0048 (16)
C9	0.0383 (19)	0.0320 (18)	0.0374 (19)	0.0025 (15)	0.0101 (15)	0.0024 (15)
C10	0.048 (2)	0.048 (2)	0.041 (2)	0.0070 (18)	0.0107 (18)	−0.0025 (17)
C11	0.055 (2)	0.053 (2)	0.050 (2)	0.006 (2)	0.020 (2)	−0.0059 (19)
C12	0.048 (2)	0.060 (3)	0.071 (3)	0.016 (2)	0.023 (2)	−0.005 (2)
C13	0.041 (2)	0.053 (2)	0.057 (3)	0.0109 (19)	0.0073 (19)	0.001 (2)
C14	0.0317 (18)	0.046 (2)	0.042 (2)	0.0060 (16)	0.0003 (16)	0.0062 (17)
C15	0.055 (2)	0.073 (3)	0.0266 (19)	0.019 (2)	0.0024 (17)	0.0043 (19)
C16	0.048 (2)	0.062 (3)	0.033 (2)	0.0135 (19)	−0.0048 (17)	−0.0018 (18)
C17	0.050 (2)	0.049 (2)	0.038 (2)	0.0106 (18)	0.0089 (17)	0.0044 (18)
C18	0.064 (3)	0.047 (2)	0.054 (3)	0.009 (2)	0.018 (2)	−0.002 (2)
C19	0.065 (3)	0.041 (2)	0.066 (3)	0.016 (2)	0.032 (2)	0.012 (2)
C20	0.077 (3)	0.057 (3)	0.043 (2)	0.018 (2)	0.021 (2)	0.014 (2)
C21	0.057 (2)	0.048 (2)	0.036 (2)	0.0127 (19)	0.0097 (18)	0.0051 (17)
C22	0.064 (3)	0.052 (2)	0.040 (2)	0.003 (2)	0.0029 (19)	0.0099 (19)
C23	0.079 (3)	0.061 (3)	0.046 (3)	0.008 (2)	0.012 (2)	0.019 (2)
C24	0.045 (2)	0.048 (2)	0.066 (3)	0.0067 (19)	0.017 (2)	0.013 (2)
C25	0.053 (3)	0.050 (3)	0.067 (3)	−0.001 (2)	−0.009 (2)	0.007 (2)
C26	0.053 (2)	0.053 (3)	0.048 (2)	0.006 (2)	−0.008 (2)	0.011 (2)
C27	0.172 (10)	0.128 (7)	0.158 (9)	−0.036 (7)	0.078 (8)	−0.001 (7)

### Geometric parameters (Å, °)

**Table:**

Co1—O2	1.891 (2)	C8—C13	1.402 (5)
Co1—N1	1.892 (3)	C8—C9	1.423 (5)
Co1—N2	1.897 (3)	C8—C14	1.440 (5)
Co1—O1	1.898 (2)	C9—C10	1.410 (5)
Co1—N4	1.977 (3)	C10—C11	1.365 (6)
Co1—N3	1.995 (3)	C10—H10	0.9300
Cl1—C19	1.728 (4)	C11—C12	1.387 (6)
Cl2—C24	1.730 (4)	C11—H11	0.9300
Cl3—O4	1.342 (6)	C12—C13	1.376 (6)
Cl3—O6	1.344 (6)	C12—H12	0.9300
Cl3—O5	1.382 (7)	C13—H13	0.9300
Cl3—O3	1.391 (6)	C14—H14	0.9300
O1—C2	1.318 (4)	C15—C16	1.515 (6)
O2—C9	1.312 (4)	C15—H15A	0.9700
O7—C27	1.319 (10)	C15—H15B	0.9700
O7—H7	0.8200	C16—H16A	0.9700
N1—C7	1.282 (5)	C16—H16B	0.9700
N1—C15	1.485 (5)	C17—C18	1.373 (6)
N2—C14	1.278 (5)	C17—H17	0.9300
N2—C16	1.477 (5)	C18—C19	1.374 (6)
N3—C17	1.339 (5)	C18—H18	0.9300
N3—C21	1.344 (5)	C19—C20	1.375 (6)
N4—C22	1.334 (5)	C20—C21	1.366 (6)
N4—C26	1.337 (5)	C20—H20	0.9300
C1—C2	1.412 (5)	C21—H21	0.9300
C1—C6	1.420 (5)	C22—C23	1.365 (6)
C1—C7	1.430 (5)	C22—H22	0.9300
C2—C3	1.403 (5)	C23—C24	1.369 (6)
C3—C4	1.381 (6)	C23—H23	0.9300
C3—H3	0.9300	C24—C25	1.369 (6)
C4—C5	1.390 (7)	C25—C26	1.363 (6)
C4—H4	0.9300	C25—H25	0.9300
C5—C6	1.355 (6)	C26—H26	0.9300
C5—H5	0.9300	C27—H27A	0.9600
C6—H6	0.9300	C27—H27B	0.9600
C7—H7A	0.9300	C27—H27C	0.9600
			
O2—Co1—N1	179.33 (12)	C11—C10—C9	121.7 (4)
O2—Co1—N2	94.28 (12)	C11—C10—H10	119.2
N1—Co1—N2	85.06 (13)	C9—C10—H10	119.2
O2—Co1—O1	85.71 (10)	C10—C11—C12	121.1 (4)
N1—Co1—O1	94.95 (12)	C10—C11—H11	119.5
N2—Co1—O1	179.83 (13)	C12—C11—H11	119.5
O2—Co1—N4	87.07 (12)	C13—C12—C11	119.2 (4)
N1—Co1—N4	92.94 (13)	C13—C12—H12	120.4
N2—Co1—N4	91.30 (13)	C11—C12—H12	120.4
O1—Co1—N4	88.86 (12)	C12—C13—C8	121.3 (4)
O2—Co1—N3	89.24 (12)	C12—C13—H13	119.4
N1—Co1—N3	90.81 (13)	C8—C13—H13	119.4
N2—Co1—N3	94.37 (13)	N2—C14—C8	125.6 (3)
O1—Co1—N3	85.46 (12)	N2—C14—H14	117.2
N4—Co1—N3	173.45 (12)	C8—C14—H14	117.2
O4—Cl3—O6	117.6 (6)	N1—C15—C16	107.9 (3)
O4—Cl3—O5	104.2 (7)	N1—C15—H15A	110.1
O6—Cl3—O5	98.6 (6)	C16—C15—H15A	110.1
O4—Cl3—O3	114.2 (5)	N1—C15—H15B	110.1
O6—Cl3—O3	115.2 (5)	C16—C15—H15B	110.1
O5—Cl3—O3	103.6 (6)	H15A—C15—H15B	108.4
C2—O1—Co1	124.5 (2)	N2—C16—C15	108.2 (3)
C9—O2—Co1	125.6 (2)	N2—C16—H16A	110.1
C27—O7—H7	109.5	C15—C16—H16A	110.1
C7—N1—C15	119.9 (3)	N2—C16—H16B	110.1
C7—N1—Co1	125.3 (3)	C15—C16—H16B	110.1
C15—N1—Co1	114.8 (2)	H16A—C16—H16B	108.4
C14—N2—C16	119.9 (3)	N3—C17—C18	123.9 (4)
C14—N2—Co1	126.0 (3)	N3—C17—H17	118.1
C16—N2—Co1	114.1 (2)	C18—C17—H17	118.1
C17—N3—C21	116.5 (3)	C17—C18—C19	118.3 (4)
C17—N3—Co1	126.2 (3)	C17—C18—H18	120.8
C21—N3—Co1	117.1 (3)	C19—C18—H18	120.8
C22—N4—C26	116.2 (4)	C18—C19—C20	119.0 (4)
C22—N4—Co1	126.6 (3)	C18—C19—Cl1	120.5 (4)
C26—N4—Co1	117.2 (3)	C20—C19—Cl1	120.4 (4)
C2—C1—C6	119.3 (4)	C21—C20—C19	119.0 (4)
C2—C1—C7	122.9 (3)	C21—C20—H20	120.5
C6—C1—C7	117.8 (4)	C19—C20—H20	120.5
O1—C2—C3	117.9 (3)	N3—C21—C20	123.3 (4)
O1—C2—C1	124.1 (3)	N3—C21—H21	118.3
C3—C2—C1	118.0 (3)	C20—C21—H21	118.3
C4—C3—C2	120.9 (4)	N4—C22—C23	123.7 (4)
C4—C3—H3	119.5	N4—C22—H22	118.1
C2—C3—H3	119.5	C23—C22—H22	118.1
C3—C4—C5	121.1 (4)	C22—C23—C24	118.7 (4)
C3—C4—H4	119.5	C22—C23—H23	120.7
C5—C4—H4	119.5	C24—C23—H23	120.7
C6—C5—C4	119.2 (4)	C23—C24—C25	119.0 (4)
C6—C5—H5	120.4	C23—C24—Cl2	121.1 (3)
C4—C5—H5	120.4	C25—C24—Cl2	119.9 (4)
C5—C6—C1	121.5 (4)	C26—C25—C24	118.5 (4)
C5—C6—H6	119.3	C26—C25—H25	120.7
C1—C6—H6	119.3	C24—C25—H25	120.7
N1—C7—C1	125.7 (3)	N4—C26—C25	123.9 (4)
N1—C7—H7A	117.2	N4—C26—H26	118.1
C1—C7—H7A	117.2	C25—C26—H26	118.1
C13—C8—C9	119.7 (4)	O7—C27—H27A	109.5
C13—C8—C14	118.4 (3)	O7—C27—H27B	109.5
C9—C8—C14	121.7 (3)	H27A—C27—H27B	109.5
O2—C9—C10	118.1 (3)	O7—C27—H27C	109.5
O2—C9—C8	124.7 (3)	H27A—C27—H27C	109.5
C10—C9—C8	117.2 (3)	H27B—C27—H27C	109.5
